# Sustainable Soil Reinforcement by Maximizing Geotechnical Performance with Rice Husk Ash in Subgrade Layers

**DOI:** 10.3390/ma18040873

**Published:** 2025-02-17

**Authors:** Abdelmageed Atef, Zakaria Hossain

**Affiliations:** Graduate School of Bioresources, Mie University, Tsu 514-0102, Japan; zakaria@bio.mie-u.ac.jp

**Keywords:** soil reinforcement, rice husk ash, subgrade layers, SEM, EDS

## Abstract

Soil reinforcement using rice husk ash and cement is emerging as an effective method for enhancing geotechnical performance in subgrade layers, offering an environmentally friendly, stable, durable, and cost-efficient solution. This study investigates sustainable soil reinforcement by maximizing geotechnical performance by applying RHA in subgrade layers. Experimental evaluations were conducted using California Bearing Ratio tests, Scanning Electron Microscopy, and Energy-Dispersive X-ray Spectroscopy. The research focused on three subgrade configurations: upper, lower, and double subgrade layers, each treated with varying proportions of cement (2%, 4%, 6%) and RHA (2%, 4%, 6%). The findings demonstrated significant improvements in bearing capacity across all subgrade layers and combinations compared to untreated control specimens. Notably, the double subgrade layer with 6% RHA + 6% cement achieved the highest CBR value of 21.30 KPa, followed by the configuration with 2% RHA + 6% cement, which recorded a CBR value of 19.62 KPa. The specimen containing 4% RHA + 6% cement achieved a CBR value of 18.62 KPa. These results highlight the effectiveness of RHA as a sustainable material for enhancing geotechnical performance in soil enhancement applications.

## 1. Introduction

Enhancing subgrade layers is critical to geotechnical engineering, mainly when dealing with loose or porous soils. Geotechnical engineers are increasingly exploring sustainable techniques to ensure the stability and efficiency of infrastructure [1,2]. In modern civil engineering, sustainable soil reinforcement has gained prominence as it addresses the growing need for environmentally conscious practices alongside infrastructure development. A promising approach involves using agricultural byproducts in soil stabilization, offering the dual benefits of improving soil performance and promoting waste management [3,4]. Among these innovations, rice husk ash (RHA) has garnered significant attention for its potential to address the global challenge of rice husk waste disposal while simultaneously enhancing ground conditions as an effective soil stabilizer [5,6,7]. The world produced 782 million tons of paddy in 2018, according to FAOSTAT data from 2020 [8]. If the rice husks had been burned, the estimated 172 million tons produced that year might have made 34 million tons of RHA [9]. However, because of its underutilization, poor market value, and transportation-related logistical difficulties, this significant amount of RHA is frequently carelessly dumped in landfills, fields, riverbanks, and open spaces close to populated areas [7,10,11,12]. Because of this, uncontrolled disposal releases hazardous materials into the air, soil, and water, endangering human health. RHA is a highly effective and sustainable material for long-term soil enhancement. It offers significant improvements in mechanical strength and durability [5,13]. The stabilization process involving RHA promotes the formation of calcium-silicate-hydrate (C-S-H) and other secondary cementitious compounds, which are key to the soil’s enhanced strength and long-term resilience [14,15,16,17,18]. The literature highlights that these compounds significantly enhance weak soils’ strength and bearing capacity [15,19]. RHA has many benefits in ground improvement projects, such as lessening the environmental impact, lowering building expenses, and using less cement and lime, which are traditional solidifying ingredients [6,20,21]. Furthermore, RHA reduces cement manufacturing and waste disposal problems, which helps to limit CO_2_ emissions from subgrade layers [14,18,22].

This study, Sustainable Soil Reinforcement by Maximizing Geotechnical Performance with Rice Husk Ash in Subgrade Layers, seeks to develop ground improvement techniques that are lasting, affordable, and ecologically friendly. The growing emphasis on sustainable development has heightened the need for eco-friendly and cost-effective approaches in geotechnical engineering. While extensive research exists in the technical literature on improving the geotechnical properties of soils using admixtures such as RHA, the majority of these studies focus on mixing RHA directly with soil. This research, however, introduces a novel perspective by exploring the application of RHA through a layering technique, specifically targeting subgrade layers in pavement construction.

Unlike traditional soil stabilization methods, the layering technique offers distinct advantages, including precise control over material distribution and optimized geotechnical performance. Subgrade layers are the foundation for overlying pavement structures, and their reinforcement is critical for ensuring long-term durability and load-bearing capacity. By concentrating RHA applications in strategically placed layers, this study aims to maximize the strength, compressibility, and bearing capacity of the subgrade, thereby enhancing overall structural stability. This innovative approach addresses a gap in current technical literature, where studies on layering techniques remain limited or unexplored. The outcomes of this research could revolutionize sustainable construction practices by reducing material usage, minimizing environmental impact, and providing a practical, scalable solution for enhancing pavement performance. In the subgrade layers of our experimental program, we have sixteen sample combinations of weak expanding soils with various combinations of soil, RHA, and cement. A more comprehensive understanding of the effects of subgrade soil upper, lower, and double layers with different proportions of RHA and cement is possible. These combinations are made with varying proportions of cement and RHA, and their impact on the bearing capacity of subgrade soils is assessed using the California Bearing Ratio (CBR) test.

To gain a deeper understanding of the structural properties, Scanning Electron Microscopy (SEM) was utilized to examine specimens with the highest recorded strength, providing valuable insights into the microstructural enhancement of the soil composites. Additionally, Energy-Dispersive X-ray Spectroscopy (EDS) was conducted to investigate the crystalline phases within the stabilized soil, revealing its detailed chemical composition. This research aims to establish practical applications for light-load construction to identify suitable applications for soil-RHA-cement mixtures in light-load construction, such as low-traffic roads, small foundations, pedestrian pathways, small access roads, or parking lots with limited vehicle weight. In such cases, this mixture can help save on costs and improve sustainability. Where high CBR values are not mandatory, this will expand the applicability of stabilized clay in real-world projects.

## 2. Research Significance

The increasing global focus on sustainable development has highlighted the demand for eco-friendly and cost-efficient innovations in geotechnical engineering. While existing studies extensively explore RHA as a soil-stabilizing additive, they predominantly focus on traditional uniform mixing methods. This research introduces a novel perspective by examining the application of RHA through a layering technique specifically tailored for subgrade layers in pavement construction. This approach remains largely unexplored in the current body of literature. The proposed layering technique offers significant advantages over conventional stabilization techniques. This approach enables precise material placement by strategically incorporating RHA into specific subgrade layers, leading to marked improvements in geotechnical properties such as strength, compressibility, and bearing capacity. This targeted reinforcement enhances the structural performance of subgrade layers, which are crucial for supporting pavement systems, ensuring more outstanding durability and load resistance under traffic and environmental stresses. This study fills a critical gap in the literature by addressing the lack of research on layering techniques for soil reinforcement. The findings have the potential to transform sustainable geotechnical practices by reducing material usage, lowering construction costs, and minimizing environmental impact. Additionally, the research provides a practical and scalable framework for designing sustainable pavements, aligning with global efforts toward greener infrastructure.

Our study seeks a sustainable solution by maximizing geotechnical performance in the upper, lower-subgrade, and double-layer subgrade systems. This approach addresses environmental concerns and the need for resilient infrastructure. Additionally, what sets our study apart from most literature is its emphasis on comparing the performance of different layer configurations to analyze and compare the CBR values across upper, lower, and double-layer compaction configurations, identifying which configuration yields the optimal load-bearing capacity compared to untreated clay. Even if the CBR value is not exceptionally high, it represents an improvement in strength, resilience, and durability for clay, which typically has lower bearing capacities.

## 3. Materials and Methods

### 3.1. Materials

**Figure 1:**
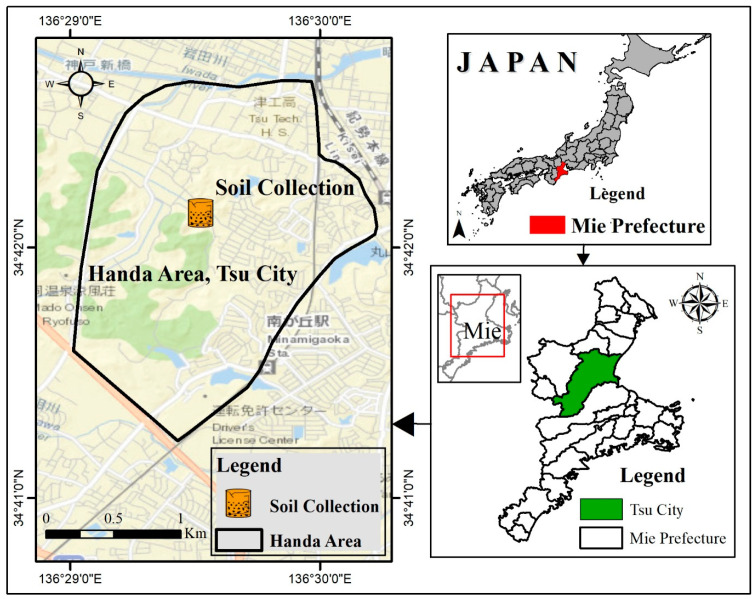
The specific location of the soil sample collection for this study.

**Figure 2:**
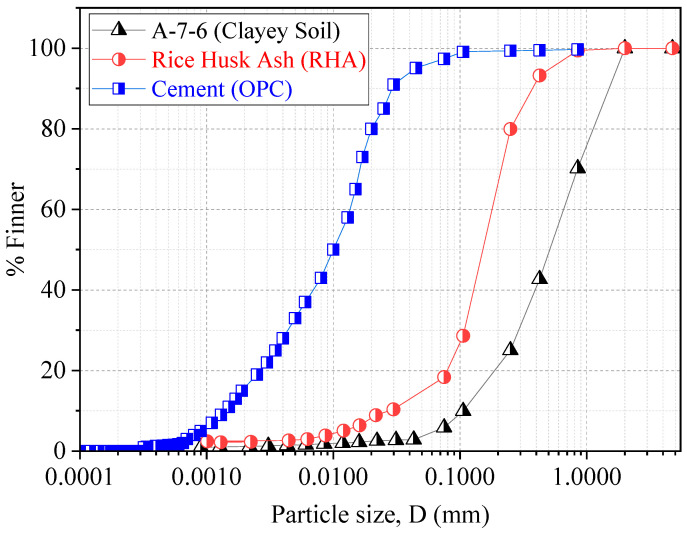
Particle size distribution for soil, RHA, and cement.

The soil for this experiment came from the Handa Area in Mie Prefecture, Japan, due to problems brought on by the poor soils in that area, as seen in Figure 1. Following three weeks of air drying [23], the soil was passed through a 2-mm sieve [24]. Afterward, analyses were performed using a sieve, a hydrometer, and a classification of the soil, liquid, and plastic limit tests. The measured constraints for plastic and liquid were 28.59% and 48.5%, respectively. The analyses showed that 42.7% of the soil was clay, 57.28% was silt, and 0.02% was sand. The American Association of State Highway and Transportation Officials (AASHTOs) categorized the soil as A-7-6 clayey. A detailed analysis of the soil, cement, and rice husk ash’s grain sizes is shown in Figure 2. The testing protocols followed the Japanese Industrial Standards JIS-A-1210 for sieve, hydrometer, liquid, and plastic limit tests [25]. Additional geotechnical features are shown in Table 1, which provides a detailed summary of the properties of the soil. Using Make Integrated Technology Co., Ltd. in Osaka, Japan, the rice husk ash used in this study was controlled by burning at 650 and 700 °C. Ash produced by this technique had a significant silica concentration of 91.10%. The rice husks were burned for 27 h in an industrial incinerator with computer controls to deliver the high silica content. The rice husk ash had particle sizes between 0.07 and 0.3 mm. Figure 2 investigates the particle size distribution of the soil and rice husk ash in detail. Table 1 provides a detailed summary of the chemical and physical properties of rice husk ash; Portland cement (OPC) was also used as a binder. The physical and chemical characteristics of OPC are shown in Table 2 [13].

### 3.2. Preparing the Specimen

To prepare the first sample for the CBR experimental samples, a saw cut was made 1 cm from the original sample to weigh the subgrade, which came out to be 600 g. Each specimen’s soil, cement, and rice husk ash mixtures were calibrated to 600 g. This mixture was well mixed in a large pan to ensure a consistent mixture of soil, RHA, and cement.

**Figure 3:**
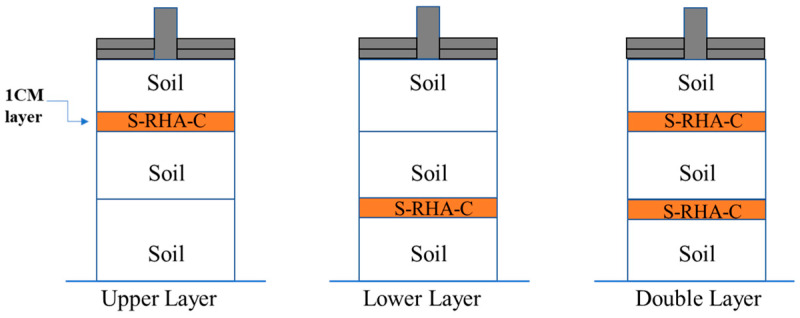
Soil-rice husk ash-cement layers.

Subsequently, water was progressively added to the dry mixture to provide proper hydration without excessive saturation. The optimal moisture content (OMC), selected from the compaction test, was used to calculate the appropriate amount of water for each specimen. The soil, cement, RHA, and water were well mixed, and the mixture was weighed once more to confirm 600 g. In the experimental setting, the upper and lower layers of the chosen subgrade layer received 600 g of the soil-RHA-cement combination. Still, the double layers received 600 g twice. The remaining strata were made entirely of soil only. The CBR test specimens were made using steel molds with internal diameters of 15 cm and a height of 17.5 cm. This systematic method prepared every specimen with precision and uniformity for the subsequent CBR testing. Next, the bottom plate, spacer disk, and mold extension were inserted after the mixture had been placed into the mold. Three soil-cement-RHA layers were formed, and each layer was tamped 67 times with an automatic rammer. This rammer measured 5.0 cm in diameter, 4.5 kg in weight, and 45.0 cm in descent height, making it a colossal tool. Following that, subgrade layers were erected inside the strata. The subgrade stratum comprised 2%, 4%, 6% cement, 2%, 4%, and 6% RHA. As shown in Figure 3, each subgrade layer was flattened using a small rammer to achieve a height of 1 cm.

Based on earlier studies [7,13,26], which recommended using amorphous silica at lower percentages, this study chose RHA dosages of 2%, 4%, and 6%.

The study’s primary goal is to improve soil by optimizing the amounts of Ca+ ions in pozzolanic processes through the controlled infusion of cement. This methodical approach helps achieve its objectives by enhancing soil qualities, highlighting environmental preservation and lowering carbon emissions which are two of the study’s main objectives. This all-encompassing approach to soil remediation goes beyond improving concrete and strives for complete soil reinforcement.

### 3.3. Testing Methods

#### 3.3.1. Compaction Tests

**Figure 4:**
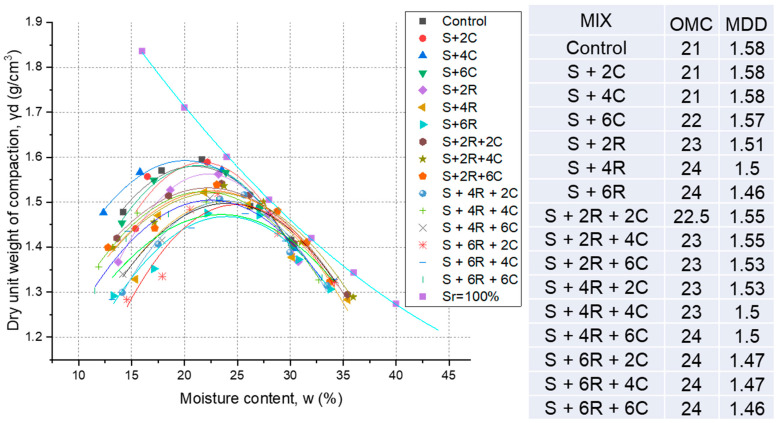
Analyzing compaction curves at different mixture ratios.

For each material mix listed in Table 3 [7], an orthogonal design of experiments with two layers and three levels was employed, ensuring minimal correlation between the last two columns (correlation = 0) to optimize the evaluation of soil stabilization. As supported by the methodologies in [27], a standard proctor compaction test was performed to ascertain the matching maximum dry density (ρ_dmax_) and optimal moisture content (w_opt_). A 2.5-kg rammer dropped from a height of 30 cm was used to compact each blend into a cylindrical mold that was 10 cm in diameter and 12.73 cm in height. The compaction effort was kept constant for each of the three layers. The dry densities were computed after repeating this process for different known moisture contents. The highest dry density and ideal moisture content were found using the compaction curves showing the link between dry density and moisture content. The Japanese Industrial Standards JIS-A-1210 were followed in every testing procedure [25]. Figure 4 presents the ideal dry density and moisture content for the soil and the soil blends.

#### 3.3.2. California Bearing Ratio Test

**Figure 5:**
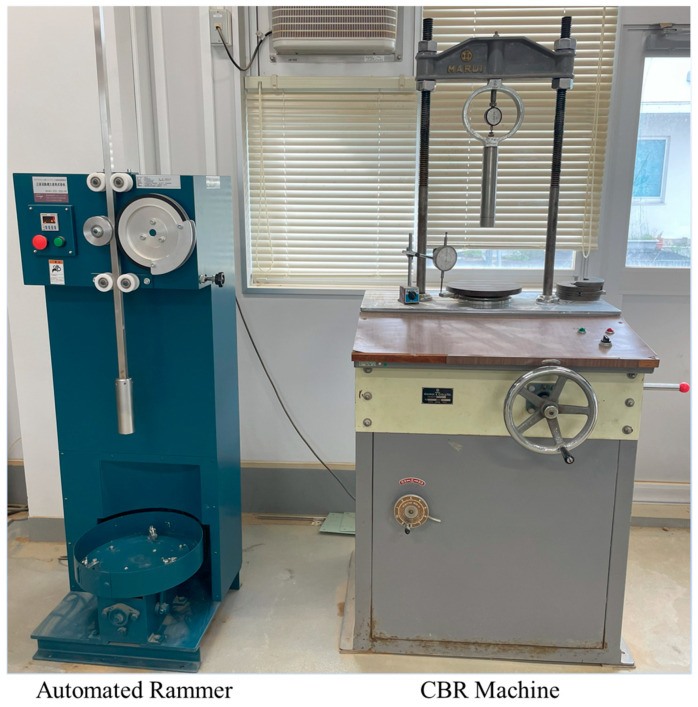
CBR test apparatus.

The CBR test assesses road subgrade and pavement areas’ bearing capacity and mechanical strength [21,28,29]. CBR values are widely used to evaluate the strength of base and sub-base materials in pavement design [18,30]. This study performed all CBR tests per the Japanese Industrial Standards JIS-A-1211 [31]. The specimens were prepared using steel molds with an internal diameter of 15 cm and a height of 17.5 cm. For each mixture, soil-RHA-cement was thoroughly blended. The specimens were compacted in three layers, receiving 67 blows from an automatic rammer. The rammer had a 5.0-cm diameter, weighed 4.5 kg, and dropped from a height of 45.0 cm, as shown in Figure 5. The specimen was then carefully positioned on the bottom plate, which held the mold and specimen. An adjustable top crosshead was used to maintain the necessary gap between the split surcharge weights inside the mold and the plunger, allowing the plunger to make contact with the upper surface of the specimen without additional loads. The tests were carried out using a standard plunger that moved at a constant speed of 1.27 mm/min (0.05 in/min) under continuous mechanical pressure from a screw jack. The load (in pounds) was recorded using a proving ring dial gauge. In contrast, a separate dial gauge measured the penetration depths of 0.5 mm, 1.0 mm, 1.5 mm, 2.0 mm, 2.5 mm, 3.0 mm, 4.0 mm, 5.0 mm, 7.5 mm, 10.0 mm, and 12.5 mm into the specimen during the operation of the CBR testing machine (Murui, Co., Ltd., Osaka, Japan). The CBR values obtained from the tests were calculated using the relevant equation:

(1) $$CBR(\%)=\frac{Load\;Strength}{Standra\;Load\;Strength}\times 100$$

#### 3.3.3. Microstructural Analysis of Soil-Rice Husk Ash-Cement Specimens

Microstructural analysis techniques were essential for thoroughly investigating and determining the bearing capacity in the reinforced and treated soil composite. In particular, we utilized Scanning Electron Microscopy and Energy-Dispersive X-ray Spectroscopy (JEOL Ltd., Tokyo, Japan). SEM was a vital tool for examining the soil composite at the micrometer level, using low-energy secondary and backscattered electrons to reveal structural changes within the newly formed composite material [3,32,33]. The three-dimensional images produced by SEM effectively illustrated how additives influenced the surface morphology of the stabilized soil on a microscopic scale [13,34,35]. Meanwhile, EDS was crucial for identifying crystalline structures and providing an in-depth analysis of how chemical stabilizing agents were incorporated into the treated soil. EDS also enabled a better understanding of the chemical transformations occurring within the treated soil, particularly those resulting from pozzolanic reactions [36,37].

## 4. Results and Discussion

### 4.1. Effects of Blends on Compaction Characteristics

Moisture content and dry density greatly influence the geotechnical performance of soil and soil mixtures, including properties such as bearing capacity. Figure 4 presents the results of the OMC and MDD for the untreated soil and various soil blends studied. The compaction curves in the figure do not intersect the saturation line (Sr = 100%), which serves as a theoretical reference to validate the relationships between OMC and MDD. This confirms that the compaction curves are physically feasible, as they remain below the saturation line, ensuring the accuracy of the results. Figure 4 shows that the moisture content increased from 21% for the untreated soil to 22%, 24%, and between 22% and 24% for the soil blends containing 6% cement, 6% RHA, and RHA-cement combinations, respectively. For example, moisture content remained at 23% and 24% for most RHA and cement combinations, except for the 2R2C blend, which showed a slightly lower moisture content of 22.5%. Meanwhile, the dry density exhibited minor variations, with values of 1.55 g/cm^3^ for the 2R2C and 2R4C blends, 1.50 g/cm^3^ for the 4R4C and 4R6C blends, and 1.46 g/cm^3^ for the 6R and 6R6C blends. The increase in moisture content can be attributed to two primary factors: the RHA high water absorption capacity due to its porous structure and the water demand associated with the heat produced due to cement hydration. Additionally, the vacancy ratio significantly increased due to changes in volume in the aggregates’ structure brought about by adding cement and RHA and, consequently, higher moisture content. Furthermore, this reduction in MDD suggests that lower compaction energy is required for these stabilized blends, which can lead to more economical compaction costs in practical applications. They found similar MDD and OMC aberrations in their experiments on soil strengthened with RHA cement [10,13].

Figure 4 highlights the significant impact of RHA and cement on the compaction characteristics of the soil. The increase in moisture content and the decrease in dry density reflect the changes in the soil’s microstructure and the improved workability of the stabilized blends. These findings underscore the potential of RHA and cement as effective stabilizing agents for enhancing the geotechnical properties of soil while offering economic benefits in terms of reduced compaction energy requirements. The observed trends in moisture content and dry density further validate the effectiveness of RHA and cement in modifying soil properties, making them viable options for sustainable soil stabilization in engineering applications.

### 4.2. Effect of Cement-Only Addition on Soil California Bearing Ratio (CBR) Values in the Subgrade Layers

**Figure 6:**
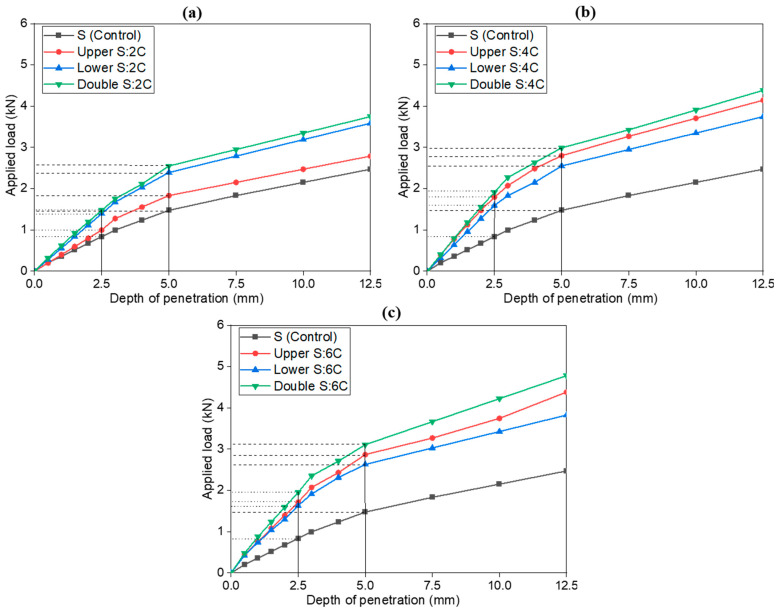
Load penetration curves of soil control, 2% C (**a**), 4% C (**b**), and 6% C (**c**), in upper, lower, and double subgrade layers.

Figure 6 illustrates the effect of adding different percentages of cement only (2%, 4%, and 6%) to clay soil on the CBR value, displaying the relationship between penetration depth (mm) and applied load (kN). The curves reveal that as penetration depth increases, the applied load increases, with higher cement content generally showing superior load resistance. We analyzed the correlation between cement content and penetration depth to further explore this relationship. The results indicate a strong positive correlation whereby increasing cement content reduces penetration depth for a given applied load. Compared to the control soil, adding 2% cement results in a moderate improvement in load-bearing capacity across all penetration depths, while 4% cement yields a noticeably higher applied load, suggesting a more substantial enhancement of soil strength. The 6% cement mixture exhibits the highest load-bearing values at every penetration depth, with CBR values of 14.41 KPa for the upper layer, 13.21 KPa for the lower layer, and 15.61 KPa for the double layer (see Table 4), indicating that 6% cement is optimal within this range.

This linearity underscores how higher cement content amplifies resistance—e.g., the 6% cement mixture reduces penetration depth by 22–35% compared to the control at equivalent loads. While formal statistical metrics were not calculated, the inverse relationship between cement content and penetration depth is visually evident and consistent across all layer configurations. For instance, increasing cement from 2% to 6% decreases penetration depth by 2.4 mm (upper layer) and 4.2 mm (double layer) at 10 mm penetration depth. Though no plateau is observed within the tested range (up to 12.5 mm), the persistent linearity beyond 5 mm suggests sustained strain-hardening behavior, characteristic of well-stabilized soils. Future work could extend penetration depths to identify plateau thresholds and employ regression models to quantify correlations more rigorously. Overall, these consistent trends across the configurations underscore the effectiveness of double-layer stabilization, which achieves the most remarkable load-bearing capacity through the combined reinforcement of both layers.

### 4.3. Effect of 2% Rice Husk Ash (RHA) and Cement on Soil California Bearing Ratio (CBR) Values in the Subgrade Layers

**Figure 7:**
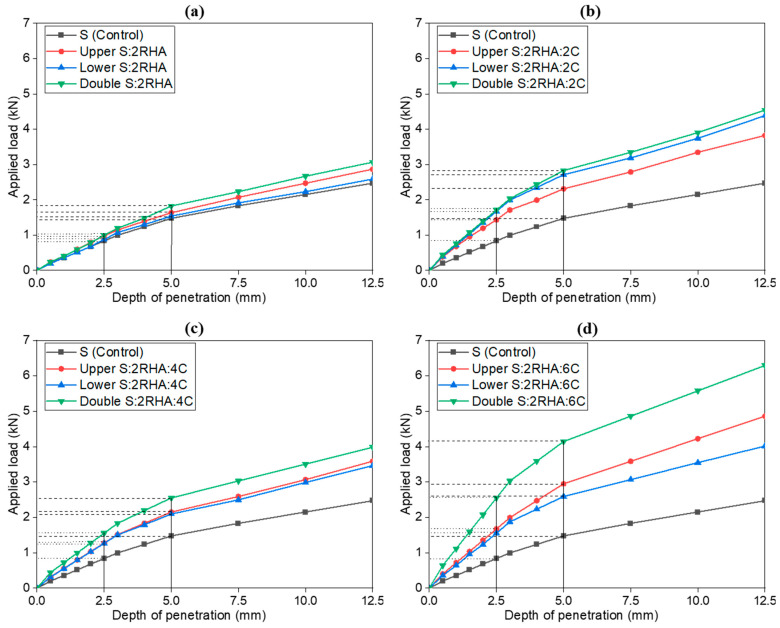
Load penetration curves of soil control, 2% RHA (**a**), 2% RHA + 2% C (**b**), 2% RHA + 4% C (**c**), and 2% RHA + 6% C (**d**), in upper, lower, and double subgrade layers.

Figure 7 shows the load-penetration curves derived from the CBR tests for the subgrade upper, lower, and double layers with 2% RHA and different cement percentages. The results demonstrate a clear trend as the cement content increases, the load required to achieve a specific penetration depth rises significantly. This behavior is consistent with the expected mechanical response of stabilized soils, where cement content with RHA enhances soil stiffness and load-bearing capacity. The load-penetration curves for all subgrade configurations (upper, lower, and double layers) display the typical CBR test trend, with a consistent linear relationship emerging beyond penetration depths of 5 mm. This linear behavior is particularly evident in blends such as the 2% RHA with 6% cement mixture, as shown in Figure 7d compared to the 2% RHA-only sample shown in Figure 7a. For example, increasing the cement content in the 2% RHA blend to 2RHA6C reduces the penetration depth by 2.2 mm in the lower layer and 5.4 mm in the double layer at a penetration depth of 10 mm. The uniformity of this linear trend across all tested cement-RHA combinations beyond the 5 mm threshold confirms the reliability of the results.

Adding 2% cement to the 2% RHA mixture results in a modest improvement over RHA alone, enhancing the bonding and reducing soil plasticity. The double-layer configuration shows the highest strength, indicating that combining cement and RHA can improve soil stability. This improvement is attributed to the synergistic effect of RHA and cement, where RHA acts as a pozzolanic material. With 4% cement, the soil’s load-bearing capacity increases more noticeably compared to the 2% cement mix. The correlation analysis further supports this finding, revealing that the 2% RHA + 6% cement combination shown in Figure 7d significantly reduces penetration depth, thereby maximizing load-bearing capacity. The upper, lower, and double configurations show improved performance, with the double layer consistently yielding the highest load capacity. This trend highlights the synergistic effect of RHA and cement when used together in layers. The load-bearing capacity reaches its peak for the 2% RHA + 6% cement combination, with the double layer achieving a significant CBR value of 19.62 KPa, compared to 14.81 KPa for the upper layer and 13.01 KPa for the lower layer. In contrast, the control soil exhibits a much lower CBR value of 7.40 KPa, as shown in Table 3. Some research revealed that soil composites, including cement and rice husk ash, improved similarly [18,38]. These results demonstrate that the double-layer configuration is the most effective approach for enhancing soil strength, far surpassing the performance of the control soil and lower cement mixes.

### 4.4. Effect of 4% Rice Husk Ash (RHA) and Cement on Soil California Bearing Ratio (CBR) Values in the Subgrade Layers

**Figure 8:**
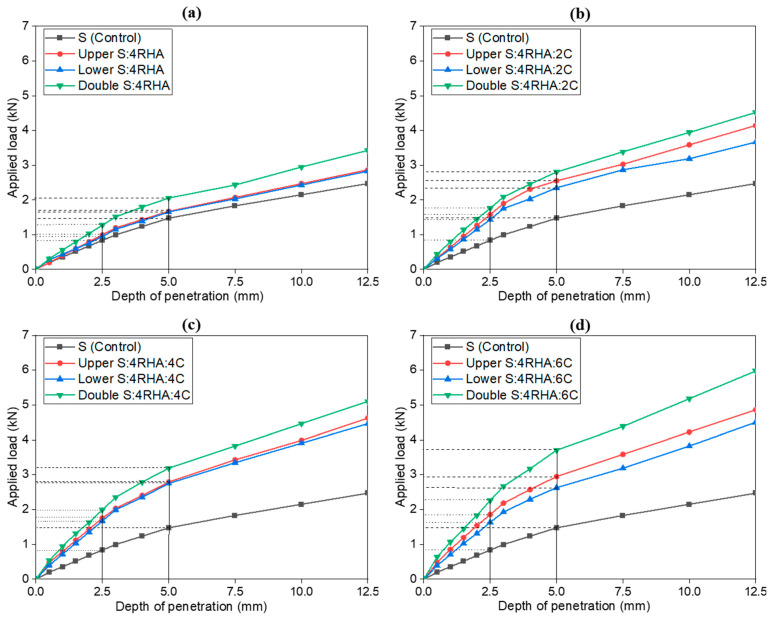
Load penetration curves of soil control, 4% RHA (**a**), 4% RHA + 2% C (**b**), 4% RHA + 4% C (**c**), and 4% RHA + 6% C (**d**), in upper, lower, and double subgrade layers.

Figure 8 explores the effect of 4% RHA with varying cement dosages on soil properties: 2%, 4%, and 6% cement. The load-bearing capacity improves compared to RHA alone, with added cement enhancing soil stiffness and reducing deformation. The CBR curves for this group show less pronounced behavior before a penetration depth of 2.5 mm, as observed mainly in Figure 8a–c. However, beyond 5 mm, all curves display similar linear trends—especially in Figure 8d—indicating consistent and reliable results. This pattern aligns with the typical response of stabilized soils, where initial penetration may vary but trends stabilize at greater depths. Our analysis of the correlation between the composition of 4% RHA with 2%, 4%, and 6% cement and penetration depth across the upper, lower, and double layers reveals a strong inverse relationship with higher cement content results in lower penetration depth. For example, in the 4% RHA blend, increasing the cement content to 6% (4RHA6C) reduces penetration depth by 2.4 mm in the upper layer and by 5.1 mm in the double layer at a penetration depth of 10 mm. These consistent trends strongly support a linear dependency within the tested range and demonstrate that cement content with RHA significantly improves the soil’s resistance to deformation while enhancing its load-bearing capacity. Among the layer configurations, the double layer achieves the best performance, followed by the upper and lower layers, suggesting the benefits of cement-RHA layering. This combination yields higher applied load values across all penetration depths, indicating effective stabilization.

The double layer remains superior, demonstrating that 4% RHA and 4% cement offer substantial strength improvements. The correlation analysis further supports this finding, showing that the 4RHA6C combination shown in Figure 8d in the double layers is more than the 4RHA2C and 4RHA4C, as shown in Figure 8b,c respectively, which substantially reduces penetration depth, maximizing load-bearing capacity. This relationship highlights the strong influence of RHA cement mixture content on soil-bearing capacity and stability. The load-bearing capacity reaches its maximum for the combination of 4% RHA and 6% cement, with the double layer achieving a CBR value of 18.62 KPa, compared to 14.81 KPa for the upper layer and 13.21 KPa for the lower layer, as shown in Table 3. In most combinations, the lower subgrade layers exhibit lower CBR values than the upper and double layers, which may be because they bear less direct load, resulting in reduced compaction and strength enhancement. Additionally, the stabilization effects of cement and RHA are less pronounced in the lower layers, as they may not fully benefit from the bonding and material interaction observed in the upper and double layers. Rachmawati et al. and some researchers also observed a similar loss in bearing capacity and CBR value in the lower subgrade layers [7,18,39]. This demonstrates that the double-layer configuration is the most effective, reflecting the synergistic effect of RHA and cement in stabilizing the soil and improving its strength properties.

### 4.5. Effect of 6% Rice Husk Ash (RHA) and Cement on Soil California Bearing Ratio (CBR) Values in the Subgrade Layers

**Figure 9:**
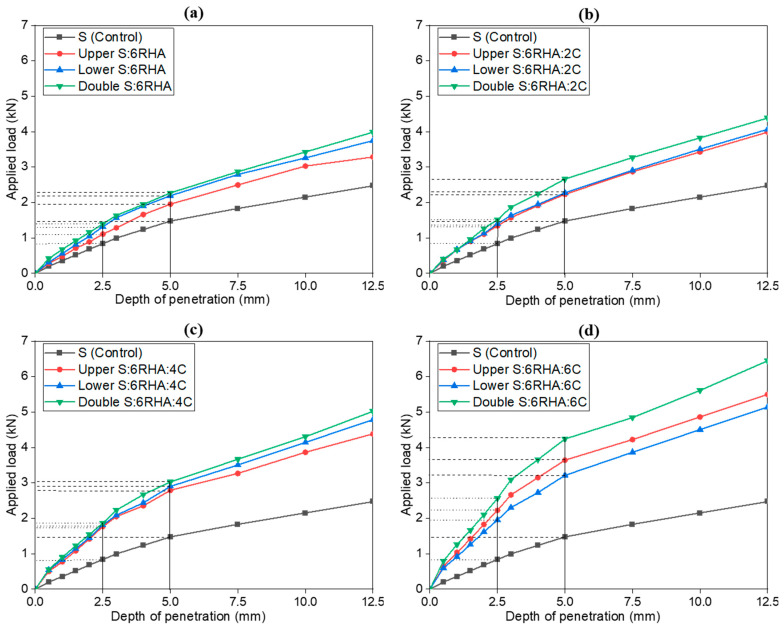
Load penetration curves of soil control, 6% RHA (**a**), 6% RHA + 2% C (**b**), 6% RHA + 4% C (**c**), and 6% RHA + 6% C (**d**), in upper, lower, and double subgrade layers.

Figure 9 presents the load-penetration curves obtained from the CBR tests for the subgrade upper, lower, and double layers stabilized with 6% RHA and varying cement percentages of 2%, 4%, and 6%. The results indicate a consistent pattern as the cement content increases; the load required to reach a specific penetration depth increases significantly. This pattern aligns with the expected mechanical behavior of stabilized soils, where the combination of cement and RHA enhances soil stiffness and load-bearing capacity. All curves follow the typical CBR test pattern, with linear trends becoming evident after a penetration depth of 5 mm, particularly in the 6RHA6C combination, as illustrated in Figure 9d. This consistency further validates the reliability of the results.

A correlation analysis between the composition of 6% RHA with 2%, 4%, and 6% cement and penetration depth in the upper, lower, and double layers reveals a strong inverse relationship. As an illustration, in the 6% RHA blend, increasing the cement content from the 6RHA2C to the 6RHA6C formulation reduces the penetration depth by 3.8 mm in the lower layer and by 5.6 mm in the double layer at a penetration depth of 10 mm. This result demonstrates enhanced resistance to deformation and improved soil strength. The CBR curves for this group exhibit less pronounced behavior at penetration depths below 2.5 mm, mainly in Figure 9b,c. However, beyond 5 mm, most curves display similar linear trends, especially in Figure 9c,d, confirming the consistency and reliability of the results. This behavior reflects the typical response of stabilized soils, where initial penetration may vary, but the trends stabilize at greater depths.

The control soil without RHA or cement consistently exhibits the lowest load-bearing capacity, while adding RHA and cement significantly enhances soil performance, with variations depending on the layer configuration. At 2% cement, the load-bearing capacity modestly improves compared to the control soil, with the double layer achieving the highest values, followed by the upper and lower layers. Increasing the cement content to 4% leads to a more noticeable improvement, as the double-layer configuration continues to outperform the upper and lower layers, reflecting the stabilizing effect of cement and RHA. Adding 6% RHA + 6% cement delivers the maximum improvement in load-bearing capacity across all tested combinations. The double-layer configuration achieves the highest value of 21.30 KPa, followed by the upper layer with 18.59 KPa and the lower layer with 16.14 KPa, as shown in Table 3. This confirms that the double-layer approach optimizes strength by combining the stabilization effects across both layers. The superior performance at 6% cement and 6% RHA can be attributed to the enhanced bonding, reduced porosity, and increased resistance to deformation. The fine particles of RHA fill voids in the soil matrix, while the silica in RHA reacts with the cement’s calcium to form additional binding compounds, as explained in the introduction, further improving soil cohesion. This combination yields the best load-bearing capacity observed in all tests. Other experiments also showed similar results regarding the efficacy of 6% RHA with cement in strengthening the clay soil in subgrade layers and increasing bearing capacity [10,13,21].

### 4.6. Correlation Between California Bearing Ratio (CBR) Value, Rice Husk Ash (RHA) Content, and Cement in the Upper, Lower, and Double Layers

**Figure 10:**
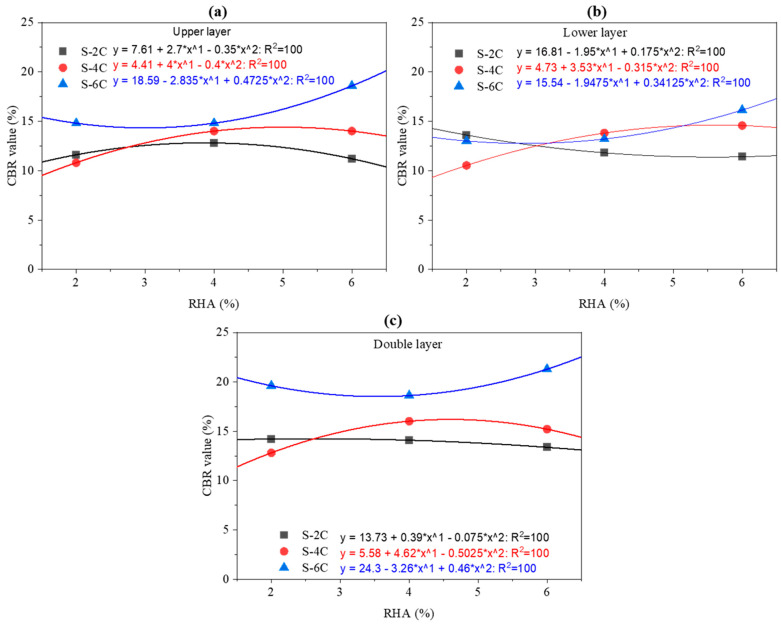
Correlation between CBR value, RHA content, and cement in the upper (**a**), lower (**b**), and double layers (**c**).

This study investigates the complex interaction between cement content, rice husk ash addition, and the mechanical performance of subgrade layers used in road construction, as quantified by the California Bearing Ratio shown in Figure 10. The research employs polynomial regression models to analyze how varying proportions of cement and RHA influence the strength of different subgrade layers, specifically the upper, lower, and double layers. These layers, defined by their position and function within the subgrade structure, exhibit unique responses to the stabilization process. For each layer, all combinations of cement and RHA percentages were systematically compared to elucidate their individual contributions to soil strength and overall stabilization performance.

In the upper subgrade layer shown in Figure 10a, a 2% cement mix initially benefits from moderate RHA incorporation, increasing CBR. However, further RHA addition eventually reduces strength, likely due to a dilution effect that impairs adequate soil particle bonding. With higher cement contents, such as 4% and 6%, the trends shift. The 4% mix reaches an optimal RHA dosage that maximizes strength before declining at higher levels. In comparison, the 6% mix demonstrates a delayed but ultimately significant improvement in CBR, suggesting enhanced pozzolanic activity and long-term stabilization benefits.

The lower subgrade layer presents a different behavior, as shown in Figure 10b. When stabilized with 2% cement, introducing RHA initially results in a slight decrease in CBR, with improvements emerging only at higher RHA dosages. This pattern hints at a delayed pozzolanic reaction. At 4% cement, there is an evident threshold beyond which additional RHA leads to diminished strength. In contrast, the 6% cement mix initially suffers a reduction in CBR but ultimately compensates with improved strength at higher RHA levels.

The double layer, which represents a combined subgrade structure integrating characteristics of the upper and lower layers, corroborates these findings, as shown in Figure 10c. Minimal strength improvements are observed with low cement content, but the soil performance markedly improves when the mix is optimized with higher cement and a balanced RHA dosage. Overall, the regression models underscore that excessive RHA without sufficient cement can compromise soil integrity, while an optimal blend enhances immediate and long-term mechanical properties.

The study emphasizes the necessity of optimizing mix proportions to harness the benefits of pozzolanic reactions while mitigating potential dilution effects associated with excessive RHA. Furthermore, the correlation equations developed in this study can be used to predict the CBR value for clay-stabilized soil when the percentages of RHA and cement are between 0 and 6%. This predictive capability is particularly valuable for designing optimized subgrade reinforcement systems, as it allows for tailored stabilization approaches that account for the nuanced interactions between RHA and cement within this practical range.

Future work will validate these regression models under field conditions and investigate microstructural changes within the subgrade layers. This will refine the predictive accuracy of the results and contribute to the development of resilient, sustainable, and economically viable soil stabilization techniques tailored for modern road construction.

### 4.7. Comparing the Upper, Lower, and Double Subgrade Layers

To determine the most effective layer configuration, we systematically compare the performance of each mixture across the upper, lower, and double subgrade layers. A concise explanation highlights the key trends in CBR values across the upper, lower, and double subgrade layers. Table 4 provides a summary of the CBR values for each combination in this configuration. The results demonstrate the progressive improvement in load-bearing capacity with the addition of RHA, cement, and their combinations compared to the control soil, which has a CBR value of 7.40 KPa. The 6% RHA with 6% cement combination achieves the highest load-bearing capacity, with values of 18.59 KPa for the upper layer, 16.14 KPa for the lower layer, and an outstanding 21.30 KPa for the double-layer configuration. This confirms the synergistic effect of RHA and cement, mainly when applied in a double-layer arrangement.

A steady increase is observed across all configurations for 6% cement content with RHA. For instance, 2% RHA with 6% cement yields 19.62 KPa in the double layer, surpassing lower cement percentages’ performance. Similarly, 4% RHA with 6% cement achieves 18.62 KPa in the double-layer configuration, highlighting its effectiveness in enhancing soil strength. In most combinations, The upper layer may outperform the lower layer because it directly bears the applied load, allowing it to fully utilize the enhanced bonding and stiffness of the stabilizing agents, such as RHA and cement. Additionally, as stress dissipates with depth, the lower layer experiences reduced anxiety compared to the upper layer, leading to a lower CBR value. These results also align with other studies [10,39]. The double-layer configuration consistently outperforms the upper and lower layers across all RHA and cement combinations. The superior results can be attributed to enhanced bonding, reduced voids, and improved strength due to the interaction of the RHA silica with the cement’s calcium, which forms additional binding compounds. The combination of stabilization in the upper and lower layers ensures a more uniform stress distribution [40,41], maximizing the benefits of the added RHA and cement throughout the soil depth. This effect is maximized with 6% RHA and 6% cement, making it the most effective combination for increasing soil strength.

### 4.8. Scanning Electron Microscopy (SEM) Analysis

In this study, incorporating a mixture of rice husk ash, cement, and soil significantly impacted the failure behavior under shear forces. A detailed microstructural analysis was conducted to understand the reinforcing mechanisms and their potential to prevent the formation of weak planes within composite materials. Initially, we examined the structural characteristics of specimens containing only soil, followed by an analysis of the composite structure after introducing RHA and cement.

**Figure 11:**
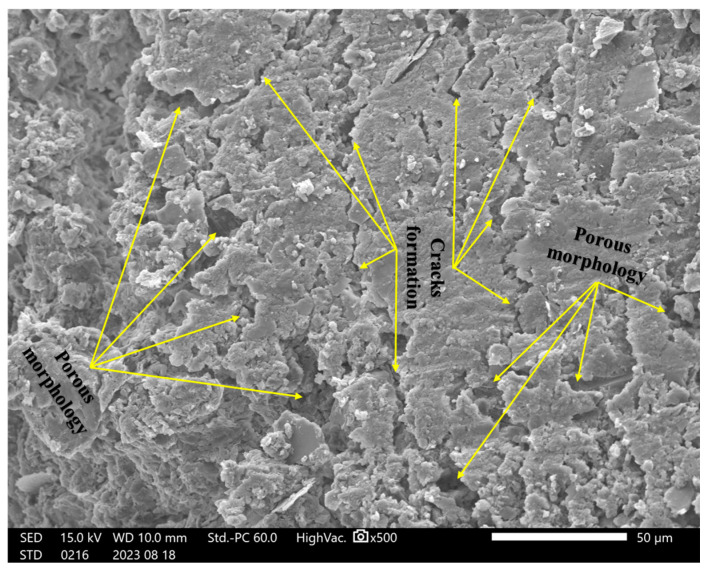
SEM analysis for soil control at ×500.

**Figure 12:**
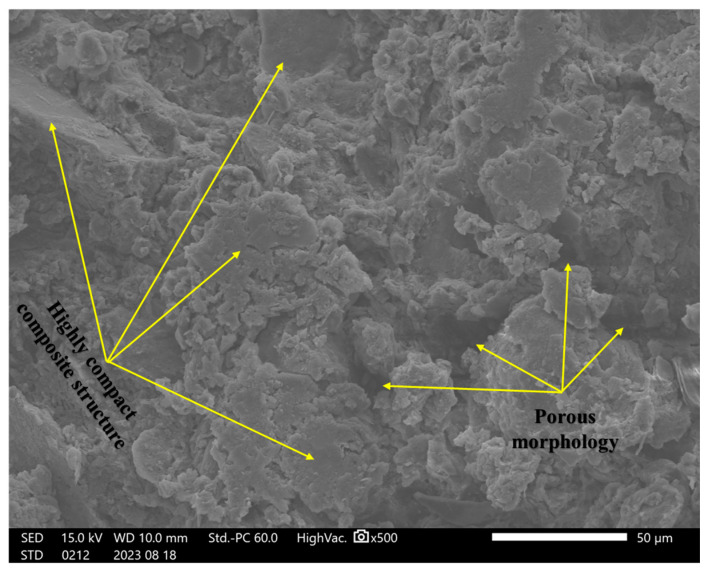
SEM analysis for 2RHA + 6C at ×500.

**Figure 13:**
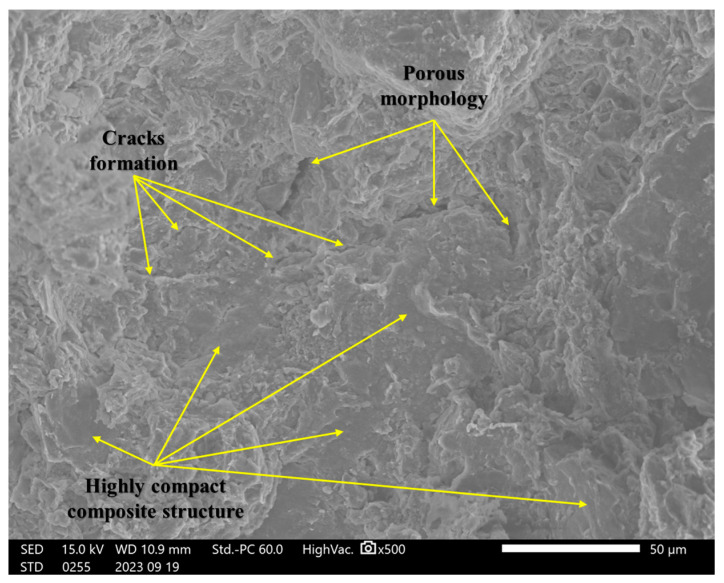
SEM analysis for 4RHA + 6C at ×500.

**Figure 14:**
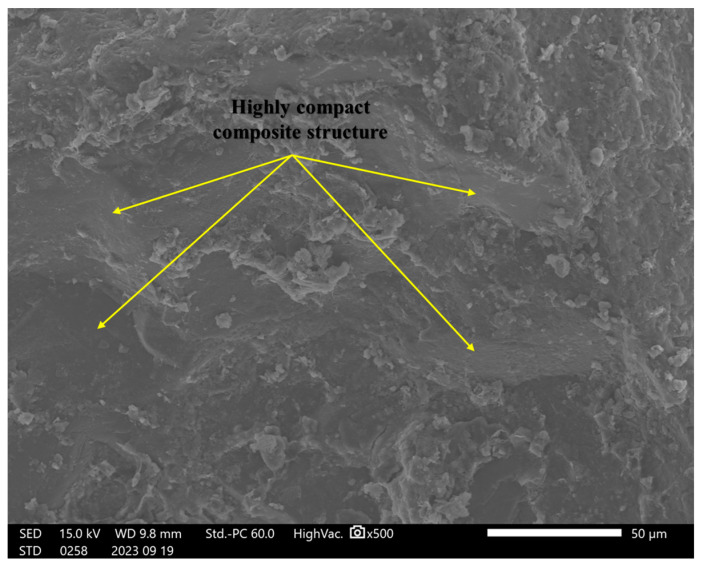
SEM analysis for 6RHA + 6C at ×500.

The microstructural analysis revealed that micro-crack formation was common in the unreinforced soil specimens. The porous structure and micro-cracks suggest a reduced resistance to deformation under loading, as the formation of weak zones within the soil matrix contributes to a lower load-bearing capacity, as shown in Figure 11 for the control sample (S Control). In contrast, the samples containing (2RHA + 6C) and (4RHA + 6C), depicted in Figure 12 and Figure 13, respectively, exhibited a notable reduction in micro-crack formation. These samples developed a more cohesive and cemented composite structure. Furthermore, adding a higher amount of RHA and cement (6RHA + 6C) significantly enhanced the degree of cementation within the composite structure, decreasing the development of micro-pores and micro-cracks, as illustrated in Figure 14. These findings are consistent with other relevant studies [7,21,42,43].

### 4.9. Mechanism of Cement Reinforcement and Rice Husk Ash (RHA)

The addition of cement and rice husk ash to clay soils considerably impacted the bearing capacity under load, as this study described. A detailed comprehension of the reinforcement mechanism was required to demonstrate their ability to stop the development of planes of weakness in the composite materials. Critical insights into the microstructural and compositional changes the RHA and cement brought were uncovered through in-depth Scanning Electron Microscopy and Energy-Dispersive Spectroscopy examinations of the reinforcement mechanism. SEM images showed how the reinforced specimens’ interfacial bonding and structural cohesion gradually developed. At first, untreated soil samples showed a porous morphology and cracks, suggesting a higher weakness in bearing capacity. However, interfacial interactions between the soil particles and cementitious chemicals quickly formed after adding RHA and cement. A more cohesive composite structure emerged, and microcrack development was significantly reduced in specimens with 2RHA + 6C and 4RHA + 6C. With higher RHA and cement content 6RHA + 6C, the improvement in microstructural integrity was further enhanced, resulting in a soil matrix that is more cemented and strong. The development of calcium-silicate-hydrate (C-S-H) and calcium-alumino-silicate-hydrate (C-A-S-H) gels as a result of the pozzolanic interactions involving RHA, cement, and soil helped to create firm and interlocking linkages, These mechanisms were also corroborated by [14,16,44,45]. Limiting particle mobility and raising the composite’s CBR values compared to control specimens dramatically boosted the bearing capacity under load within the composite.

Complementary results from the EDS analysis linked higher SiO_2_ and CaO levels to higher RHA content and the formation of C-S-H gels due to pozzolanic activity. In the C-S-H gel [13,19,46], aluminum ions improved mechanical characteristics by reinforcing the composite structure. The overall stability and endurance of the reinforced soil were enhanced by these microstructural advancements, which guaranteed proper load transfer within the composite material and successfully stopped cracks from spreading. The effectiveness of RHA and cement as reinforcing agents is demonstrated by the combined effects of enhanced particle packing, cementitious binding, and pozzolanic processes, which provide a robust and long-lasting composite material appropriate for maximizing geotechnical performance.

### 4.10. Energy-Dispersive Spectroscopy (EDS) Analysis

**Figure 15:**
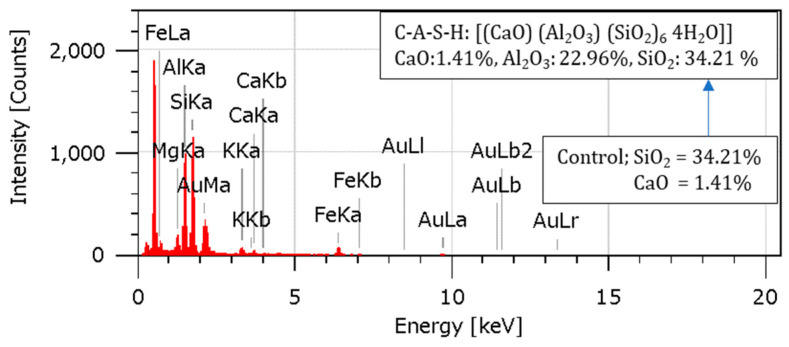
EDS analysis for soil control.

**Figure 16:**
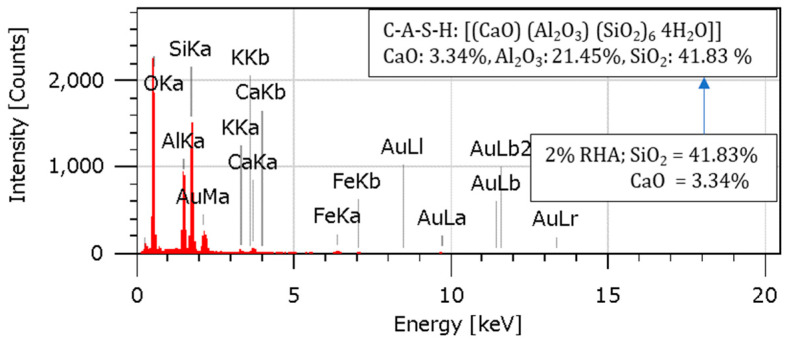
EDS analysis for 2RHA6C.

**Figure 17:**
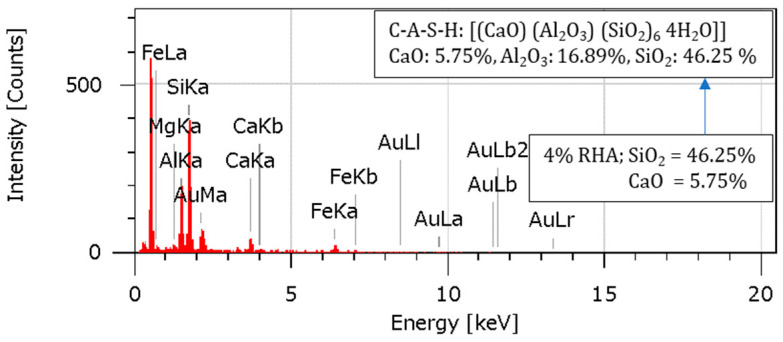
EDS analysis for 4RHA6C.

**Figure 18:**
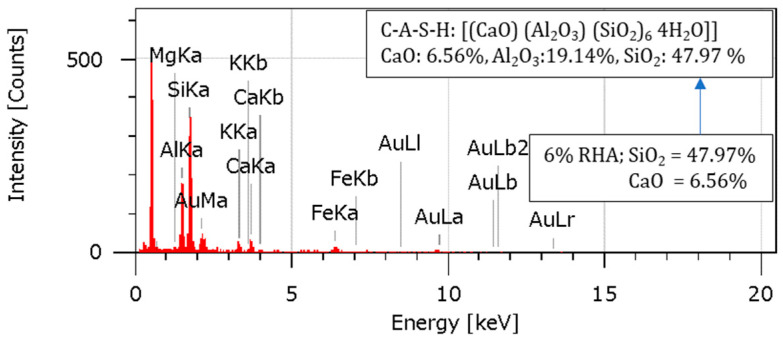
EDS analysis for 6RHA6C.

An Energy-Dispersive Spectroscopy examination was conducted to learn more about the various elements’ relative abundance and elemental composition in the specimens. The EDS spectra and elemental compositions for the soil, 2RHA + 6C, 4RHA + 6C, and 6RHA + 6C samples are shown in Figure 15, Figure 16, Figure 17 and Figure 18. The EDS spectra and elemental compositions for the untreated soil, 2% RHA + 6% cement, 4% RHA + 6% cement, and 6% RHA + 6% cement samples are presented in Figure 14, Figure 15, Figure 16 and Figure 17. The results reveal that the chemical composition of the samples was significantly influenced by the addition of cement and RHA as stabilizing agents. RHA, being rich in silica (SiO_2_), contributed to a noticeable increase in SiO_2_ content in the stabilized samples—similarly, cement added calcium oxide (CaO) into the soil matrix. As the RHA content increased from 2% to 6%, the EDS spectra showed a corresponding rise in SiO_2_ and CaO levels. This trend highlights the synergistic effect of RHA and cement in altering the soil’s chemical composition, which is critical for improving its mechanical properties.

The pozzolanic reaction between RHA, cement, and soil led to the formation of calcium-silicate-hydrate (C-S-H) gel, a primary binder responsible for enhancing soil strength. The presence of aluminum ions further accelerated this reaction [7,47,48]. Stronger calcium-alumino-silicate-hydrate (C-A-S-H) bonds were formed due to the uptake of aluminum ions on the gel’s surface and inside its interlayer. As seen in Figure 16 and Figure 17, these bonds, known as Calcium Alumino Hexasilicate Tetrahydrate [(CaO)(Al_2_O_3_)(SiO_2_)_6_ 4(H_2_O)], help to increase the composite material’s strength and stability.

The EDS results provide valuable insights into the mechanisms underlying the stabilization process. The increased SiO_2_ and CaO content, coupled with the formation of C-S-H and C-A-S-H gels, explains the enhanced mechanical properties of the stabilized soil. These findings align with previous research, which has consistently shown that the combination of RHA and cement effectively improves the geotechnical performance of expansive soils [13,49,50]. While the current study demonstrates the effectiveness of RHA and cement in soil stabilization, further research could explore the long-term durability of these stabilized soils under varying environmental conditions, such as wet-dry cycles. Additionally, the potential use of other pozzolanic materials in combination with RHA and cement could be investigated to optimize the stabilization process further.

According to the EDS analysis, the addition of RHA and cement significantly alters the soil’s chemical composition and microstructural properties, leading to improved strength and stability. These findings validate the efficacy of RHA and cement as sustainable and effective stabilizing agents for expansive soils.

## 5. Conclusions

This study thoroughly examined the impact of incorporating rice husk ash and cement into clay soils for use as subgrade layers. The investigation included California Bearing Ratio tests, Scanning Electron Microscopy, and Energy-Dispersive X-ray Spectroscopy to evaluate the stabilized soil’s mechanical and microstructural properties. The results demonstrated a significant enhancement in bearing capacity and microstructural characteristics due to the stabilization provided by RHA and cement. The combination of these materials substantially improved CBR values across all tested subgrade configurations, with the double-layer configuration achieving the highest CBR value of 21.30 KPa in the (6% RHA + 6% C) sample. This improvement is attributed to pozzolanic reactions and enhanced particle interlocking, which increased friction and minimized particle movement. The SEM analysis revealed microstructural changes, including forming cohesive and cemented composite structures with fewer microcracks and micropores. The EDS analysis confirmed the chemical transformations, highlighting the formation of calcium-silicate-hydrate (C-S-H) and calcium-aluminum-silicate-hydrate (C-A-S-H) bonds, contributing to the soil composite’s improved cohesion and stability.

The combination of mechanical testing and microstructural analyses underscored the effectiveness of RHA-cement stabilization in improving soil performance. This research demonstrated that incorporating RHA and cement into clay soils significantly enhances their mechanical properties and microstructural integrity. The study identified the 6% RHA + 6% C, 2% RHA + 6% C, and 4% RHA + 6% C configurations as promising sustainable solutions for soil reinforcement and improved geotechnical performance in subgrade layers. The comprehensive approach adopted in this study provides valuable insights into the material’s behavior, and further research is recommended to assess the long-term performance and environmental impact of this composite material in diverse geotechnical applications.

## Figures and Tables

**Table 1:** Characteristics of soil and RHA.

Contents	Characteristics	Amount
soil characteristics	maximum dry density, g/cm^3^	1.58
	optimum moisture content	21%
	sand (75 μm–2 mm), %	0.02
	silt (5–75 μm), %	57.28
	clay < 5 μm, %	42.7
	liquid limit, LL, %	48.5
	plastic limit, PL, %	28.594
	plasticity index, PI, %	19.906
	AASHTO classification	A-7-6(5)
RHA characteristics	average particle size, mm	0.001 to 0.3
	loss of ignition, %	4–6
	specific gravity, g/cm^3^	2.12
	burning temperature, °C	650–700
	burning time, hour	27
	silica (SiO_2_), %	91.10
	carbon dioxide (CO_2_), %	4.35
	potassium oxide (K_2_O), %	2.40
	calcium oxide (CaO), %	0.57
	iron oxide (Fe_2_O_3_), %	0.05
	alumina (Al_2_O_3_), %	0.03
	others, %	1.50

**Table 2:** Characteristics of cement.

Contents	Characteristics	Value
Cement characteristics	initial setting time, minutes	170
	final setting time, minutes	225
	specific gravity, g/cm^3^	3.15
	specific surface area, m^2^/kg	340
	28-day compressive strength, MPa	33–53
	calcium oxide (CaO), %	63.40
	silicon dioxide (SiO_2_), %	21.60
	iron oxide (Fe_2_O_3_), %	5.35
	alumina (Al_2_O_3_), %	4.45
	sulfur trioxide (SO_3_), %	1.92
	magnesium oxide (MgO), %	1.65
	sodium oxide (Na_2_O), %	0.11
	potassium oxide (K_2_O), %	0.22
	loss of ignition, %	<4

**Table 3:** Detailed blend combinations.

Mixtures	Soil, S (%)	RHA (%)	Cement, C (%)
Soil	100	0	0
S:2C	98	0	2
S:4C	96	0	4
S:6C	94	0	6
S:2R	98	2	0
S:4R	96	4	0
S:6R	94	6	0
S:2R:2C	96	2	2
S:2R:4C	94	2	4
S:2R:6C	92	2	6
S:4R:2C	94	4	2
S:4R:4C	92	4	4
S:4R:6C	90	4	6
S:6R:2C	92	6	2
S:6R:4C	90	6	4
S:6R:6C	88	6	6

**Table 4:** Values of the California bearing ratio for upper, lower, and double subgrade layers.

Mix Types	Upper	Lower	Double
Soil (Control)	7.40		
Soil + 2% Cement	9.21	12.01	12.81
Soil + 4% Cement	14.05	12.81	15.01
Soil + 6% Cement	14.41	13.21	15.61
Soil + 2% RHA	8.21	7.72	9.17
Soil + 4% RHA	8.41	8.33	10.33
Soil + 6% RHA	9.81	11.01	11.41
Soil + 2% RHA + 2% Cement	11.61	13.61	14.21
Soil + 2% RHA + 4% Cement	10.81	10.53	12.81
Soil + 2% RHA + 6% Cement	14.81	13.01	19.62
Soil + 4% RHA + 2% Cement	12.81	11.81	14.09
Soil + 4% RHA + 4% Cement	14.01	13.81	16.02
Soil + 4% RHA + 6% Cement	14.81	13.21	18.62
Soil + 6% RHA + 2% Cement	11.21	11.41	13.37
Soil + 6% RHA + 4% Cement	14.01	14.57	15.21
Soil + 6% RHA + 6% Cement	18.59	16.14	21.30

## Data Availability

The original contributions presented in this study are included in the article/Supplementary Material. Further inquiries can be directed to the corresponding author.

## Supplementary Materials

The following supporting information can be downloaded at: https://www.mdpi.com/article/10.3390/ma18040873/s1.

## Abbreviations

The following abbreviations are used in this manuscript:

RHA	Rice Husk Ash
S	Soil
C	Cement
CBR	California Bearing Ratio
SEM	Scanning Electron Microscopy
EDS	Energy-Dispersive X-ray Spectroscopy
OMC	Optimum Moisture Content
MDD	Maximum Dry Density
